# How Data Analytics and Big Data Can Help Scientists in Managing COVID-19 Diffusion: Modeling Study to Predict the COVID-19 Diffusion in Italy and the Lombardy Region

**DOI:** 10.2196/21081

**Published:** 2020-10-14

**Authors:** Davide Tosi, Alessandro Campi

**Affiliations:** 1 Università degli Studi dell'Insubria Varese Italy; 2 Politecnico di Milano Milano Italy

**Keywords:** COVID-19, SARS-CoV-2, big data, data analytics, predictive models, prediction, modeling, Italy, diffusion

## Abstract

**Background:**

COVID-19 is the most widely discussed topic worldwide in 2020, and at the beginning of the Italian epidemic, scientists tried to understand the virus diffusion and the epidemic curve of positive cases with controversial findings and numbers.

**Objective:**

In this paper, a data analytics study on the diffusion of COVID-19 in Italy and the Lombardy Region is developed to define a predictive model tailored to forecast the evolution of the diffusion over time.

**Methods:**

Starting with all available official data collected worldwide about the diffusion of COVID-19, we defined a predictive model at the beginning of March 2020 for the Italian country.

**Results:**

This paper aims at showing how this predictive model was able to forecast the behavior of the COVID-19 diffusion and how it predicted the total number of positive cases in Italy over time. The predictive model forecasted, for the Italian country, the end of the COVID-19 first wave by the beginning of June.

**Conclusions:**

This paper shows that big data and data analytics can help medical experts and epidemiologists in promptly designing accurate and generalized models to predict the different COVID-19 evolutionary phases in other countries and regions, and for second and third possible epidemic waves.

## Introduction

The unexpected pandemic diffusion of COVID-19 [1] worldwide calls for the need to study all available data to promptly understand the epidemic curve of COVID-19 contagiousness [2-4] to help medical experts, epidemiologists, and political decision makers in designing prompt reaction plans.

In this paper, we aim to develop an accurate predictive model tailored to forecast the evolution of the diffusion over time, exploiting big data and data analytics. Generally, epidemics follow an exponential curve in the spread of positive cases. This is not the case of the curve observed in Wuhan, China [5], where the official curve of confirmed positive cases of COVID-19 follows a behavior that is different from typical epidemics. More precise studies are reported in [1,6-8]. Starting from this observation, we tried at the beginning of March to correlate the Wuhan official data set with the COVID-19 data set available in Italy by applying big data and data analytics techniques [9] to all official open data available worldwide [5] to design the logistic curve for early estimation of the number of COVID-19 positive cases day-to-day and in all the phases of the pandemic over time (eg, the peak in the number of daily cases, the logistic plateau, and at the end of the pandemic).

## Methods

To design the predictive model, we exploited all the official data sets available so far. We analyzed the Wuhan official data set, available at [5]. As for the Italian perspective, we adopted the official data set that is daily published and updated by the Department of the Italian Civil Protection [10]. All these data sets are freely available. Additional statistics (eg, percentage of people that have severe COVID-19, percentage of people that need intensive care, COVID-19 mortality index) have been imported from the World Health Organization (WHO) website [11].

Exploiting the similarity between the behavior of the COVID-19 contagion in Wuhan and the starting Italian scenario, we designed our predictive curve adapting well-known mathematical methods to this particular context: the Pearson correlation index to formally evaluate this similarity in the contagion, the logistic curve (sigmoid) to design the cumulative number of COVID-19 positive cases, regression models to evaluate the best correlation fit for our predictions, and the power law to model the initial ascent of the pandemic.

## Results

In the first step of our method, we computed the Pearson correlation coefficient applied to a sample between the two data sets (Italy vs Wuhan) with reference to the daily new positive cases as:


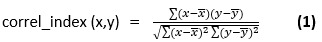


**Figure 1:**
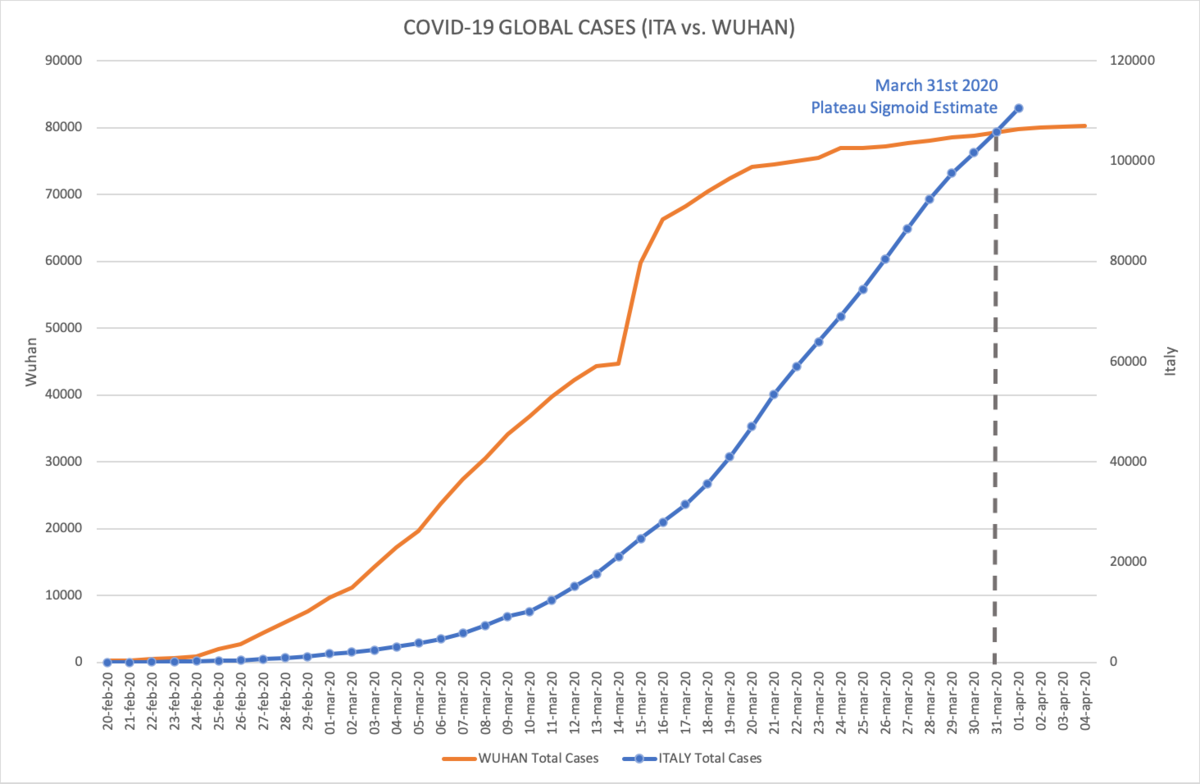
Graphical comparison between the Italian curve and Wuhan curve (total cumulative positive cases).

Starting from the data set available from March 2, 2020, it was possible to observe a strong Pearson correlation between the data set related to Italy and the data set related to Wuhan with a *Correlation Index of 0.9944,* as depicted in Figure 1 where the Wuhan curve is compared with the Italian one.

Starting from this assumption, it was then possible to use the Wuhan data set (with a strong statistical significance) to try to predict the logistic curve of total positive cases to COVID-19 for Italy and the Lombardy Region. We started designing our model by the basic assumption that every pandemic phenomenon follows a logistic (sigmoid) curve. We then searched for a curve that best fit the initial growing part of the logistic, and we found that the best fit is a power law curve in the form of y = m * x^b^. To compute the coefficients m and b of the power curve, we started our elaborations with two other assumptions in mind:

1. We based our initial estimation on the number of swab tests analyzed in the initial days of the pandemic period, where an average of 8000 swab tests were performed daily (in May, the number of swab tests was increased significantly to an average of 50,000 tests/day).
2. We assumed that the Italian Government would act promptly with restrictions and lockdowns on the Italian population and that the Italian citizens would follow these restrictions with a sense of responsibility.

Moreover, we used the stabilized data set for Wuhan City, and we adopted additional official statistics (published by the WHO [11]) about the COVID-19 spread in China (eg, percentage of severe disease cases, percentage of critical disease cases, crude fatality ratio, the number of days passed in each phase of the China pandemic) to adjust the multiplier coefficient of our predictive model. In this way, we were able to estimate the position of the first inflections of the curve (to estimate the date for the peak of new daily positive cases), the second curve inflection (to estimate the date of the curve plateau), and then to determine the day-to-day number of positive cases during the second part of the pandemic (ie, during the descent.)

**Figure 2:**
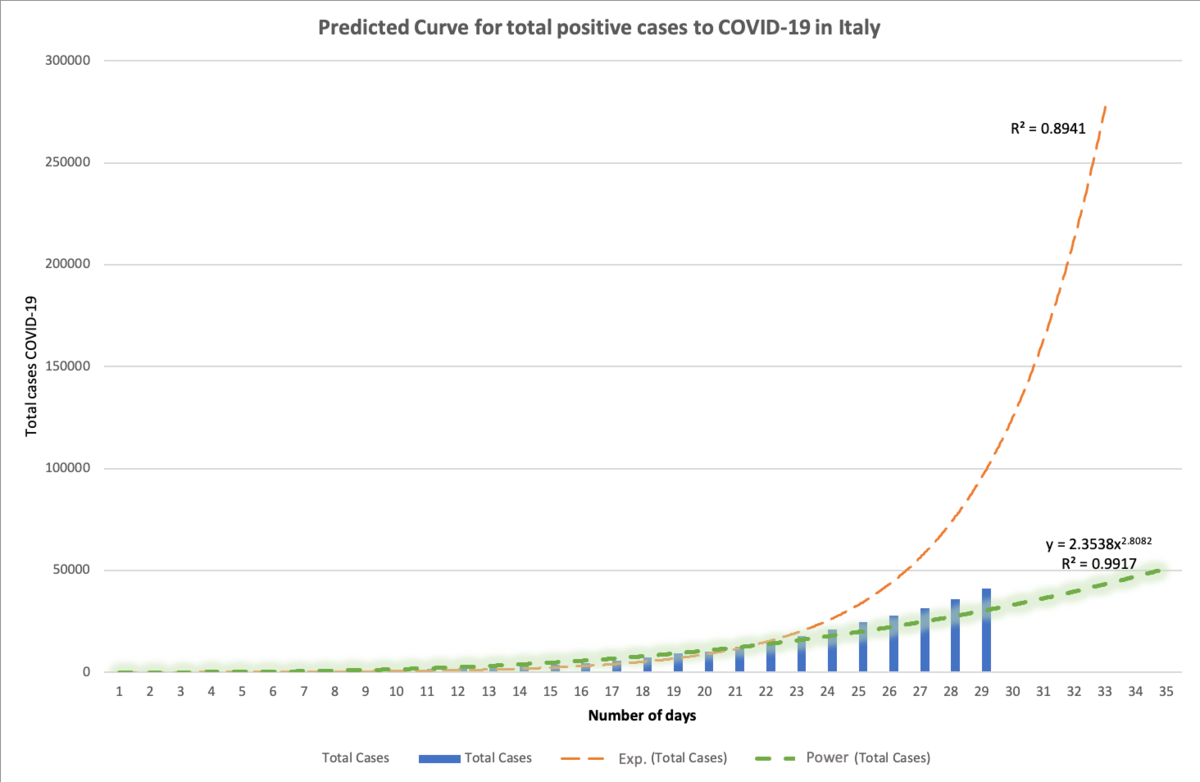
The predicted curve of total positive cases in Italy (as of March 2, 2020).

Figure 2 shows the number of new daily cases in Italy starting from the first identification of a patient in the town of Codogno (Lodi, Lombardy) on February 24, 2020. The dotted orange line is the classical exponential curve typical of epidemics, while the green line is the best fit for the data set available for Italy until March 2, that is a power curve in the form of y = 3.00 * x^2.77^.

The goodness of fit for the detected model is high, with a high coefficient of determination (*R*^2^=0.9917).

**Figure 3:**
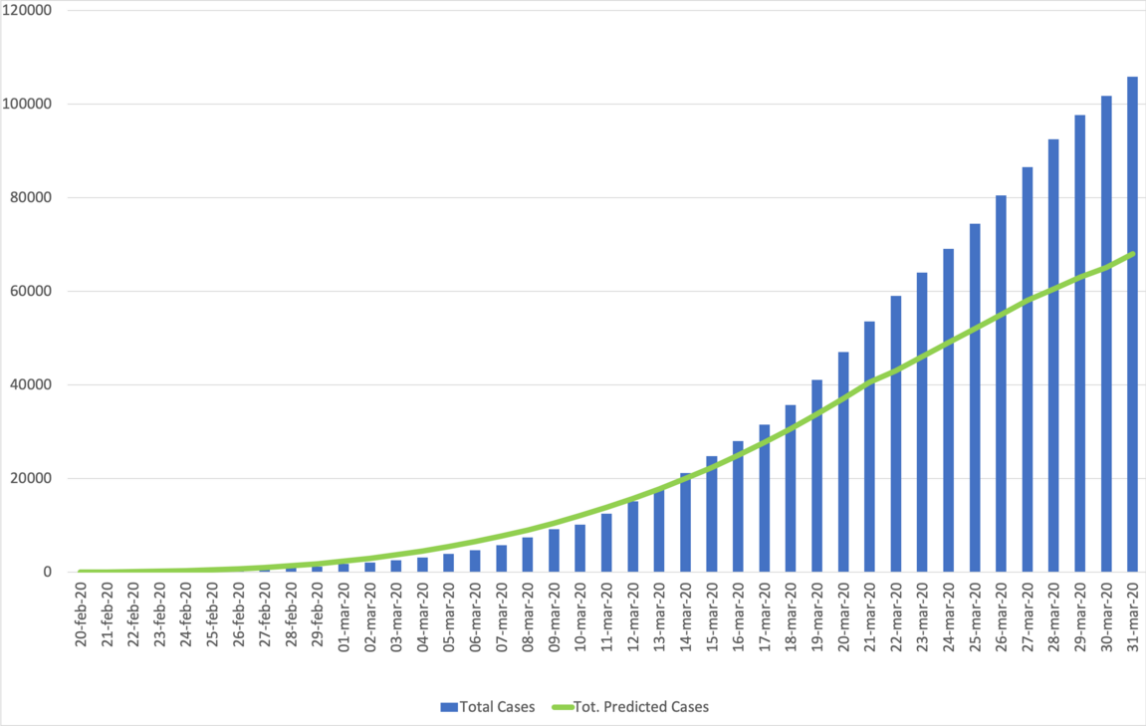
Graphical comparison between the real vs predicted number of Italian cumulative cases. Tot.: total.

Figure 3 compares the actual behavior of cumulative COVID-19 positive cases with the predictions of our model over time until the end of March (where our model predicted the logistic plateau).

Our model forecasted (18 days in advance) the peak in the number of daily new cases on March 21, 2020, with a total of 42,000 positive cases against the official datum of 53,500 total positive cases, with a confidence level of 95%. The model significantly outperformed other predictions based on exponential models that forecasted more than 180,000 positive cases.

As highlighted in Figure 3, the model underestimated some of the real official values for several reasons:

- March 21, 2020, is registered as the date with the absolute peak in the number of daily new cases, hence the error (that is a cumulative error) is increasing over time.
- The Lombardy Region has the same consideration (where the peak has been forecasted and actually observed on March 17). The Lombardy Region accounts for 50% of the overall national value.
- The predictive model is strongly related to the curve of Wuhan. Although the increase of daily cases is similar between the Italian curve and the Wuhan curve, the decrease is a bit different. The Italian one is less steep than the Wuhan decrease since the restrictions implemented by the Italian government are less stringent than the Wuhan restrictions, and the Italian population reacted to the restrictions with less determination.

**Figure 4:**
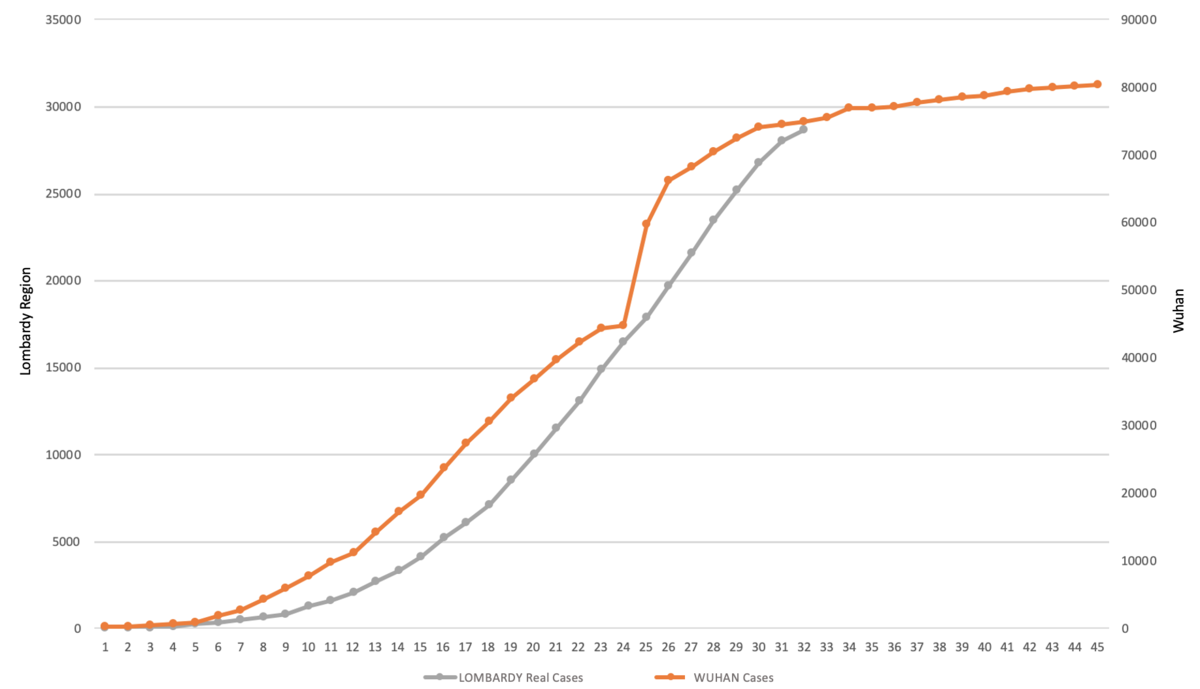
Graphical comparison between the Lombardy Region curve and the Wuhan curve (total cumulative positive cases).

Moreover, it is important to highlight that (as depicted in Figure 4) the plateau for the Lombardy Region was predicted and confirmed to start at the end of March, a few days in advance of the plateau for the Italian curve, hence the absolute contribution of the Lombardy Region on the total national value was decreasing over time. It is important to observe that the Lombardy Region anticipated all the national restrictions by 1 week with the Decree to Manage the COVID-19 Emergency: DPCM-9 March 4, 2020.

The model elaborated from the beginning of March 2020 (Figures 1-3) predicted the plateau (as confirmed by Prof S Brusaferro from Istituto Superiore Sanità during a national live TV broadcast [12]) for the logistic Italian curve at the end of March with an estimate on the total positive cases to be 68,000 (while the actual value was 100,000 total cases). It is important to highlight that the model focused on the prediction of the pandemic timeline and not on the exact number of people officially testing positive for COVID-19, a number that is well known to be dependent on the number of analyzed swab tests. We based our initial estimation on the number of swab tests analyzed in the initial days of the pandemic period. The increasing amount of swab tests moved our curves toward higher values but did not modify the prediction regarding the starting day of the logistic curve plateau and the prediction about the different phases of the COVID-19 pandemic. As previously stated, another factor that contributes to the larger number of affected people is the different enforcement and timeliness applied to the restrictions and lockdowns by the Italian government and the Italian population compared to China, which we adopted in our initial assumptions. In any case, in the forecast of the total positive cases in a pandemic phenomena, the magnitude of the values is the real discriminant on the quality of the prediction. Exponential curves (adopted by several scientists at the beginning of the COVID-19 pandemic in Italy) predicted 1 million cases for the end of March, with a ten-fold overestimation, while our model underestimated for a value less than 40%. Ricolfi [13] estimated for March 8, 2020, 60,000 total cases, while the official value was 7400, thus an overestimation of 800%. In Italy, we have 60 million inhabitants; a difference of 32,000 cases between the estimated value and the actual value on this population introduces an error that is less than 0.08%, and this does not impact the governmental policies and actions to preserve the national health system.

It is important to observe that reaching the plateau does not indicate that the COVID-19 epidemic has been solved, but it means that the cumulative number of COVID-19 positive cases is slowing down in its ascent. The WHO guidelines suggest waiting for the contagion to be +0 (ie, the new daily cases are reduced to a few dozen positive cases per day) and then taking restrictions for two additional cycles of COVID-19 incubation (mean incubation period 5-6 days, range 1-14 days.) Hence, considering the national plateau on March 31, 2020, we estimated the actual containment of the COVID-19 epidemic at the beginning of June for the following reasons:

- One additional month, after the beginning of the plateau, to reach a small and contained number of new daily cases (end of April)
- One additional month in waiting for the two COVID-19 incubation cycles (end of May)
- Gradual return to normal life starting from the beginning of June

In Figure 4, the two curves for total cases in Wuhan (orange line) and the Lombardy Region (gray line) are depicted. It is interesting to observe that in this case the two behaviors are similar and comparable. Figure 4 also shows that we are approaching the plateau for the Lombardy Region.

**Figure 5:**
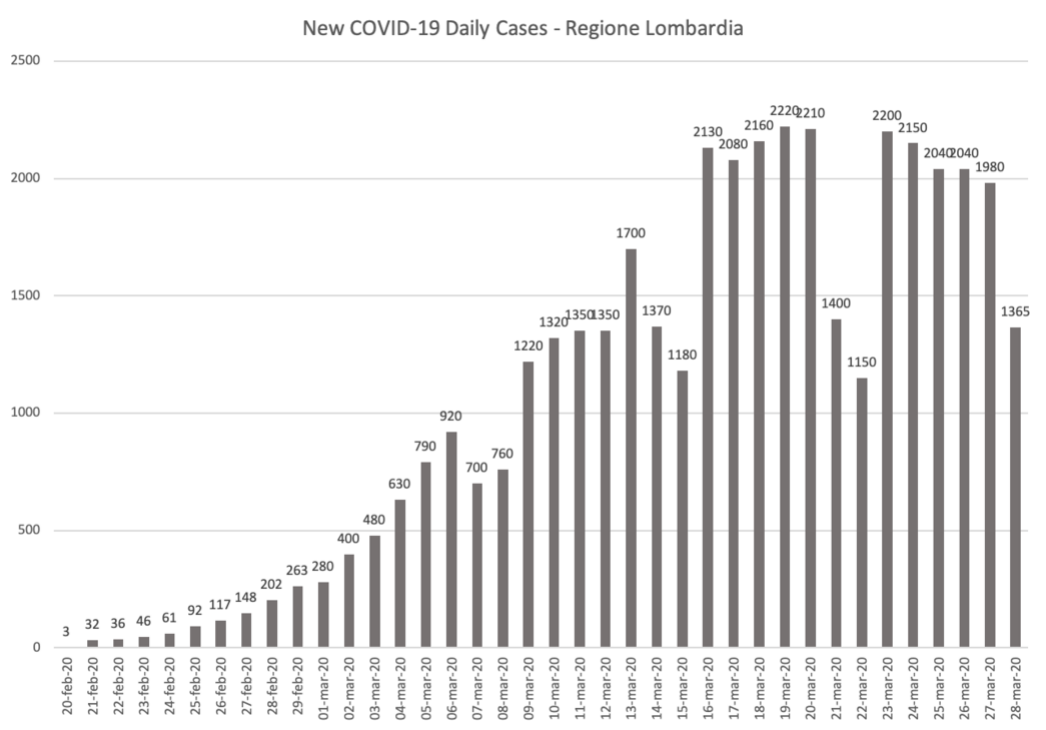
Graphical plot of daily new cases in the Lombardy Region.

**Figure 6:**
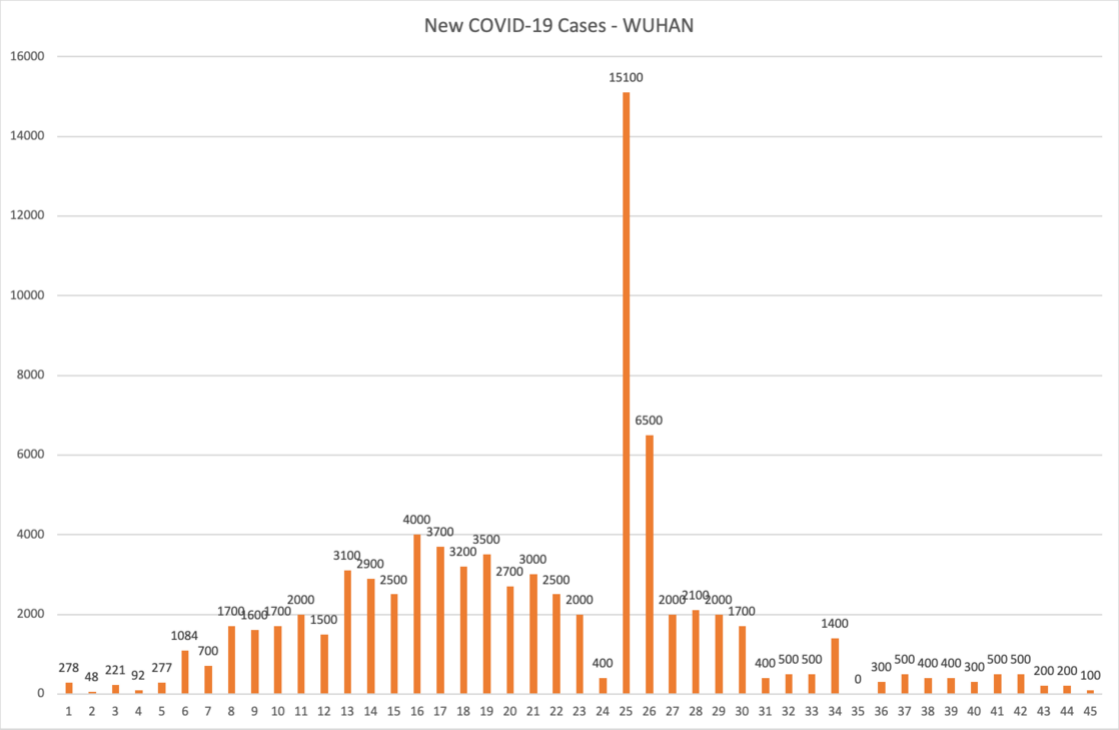
Graphical plot of daily new cases in Wuhan.

In Figures 5 and 6, we show the new COVID-19 cases per day in the Lombardy Region and Wuhan, respectively. As previously mentioned, the peak in the Lombardy Region was registered on March 17, 2020, with 2200 new daily cases; then, the number of daily cases decreased over time (with some exception as you can see in Figure 5).

This is a good indicator (the peak matches the inflection point in the logistic curve) that we had started the descent toward the plateau in the Lombardy Region (the last inflection point in the logistic curve).

**Figure 7:**
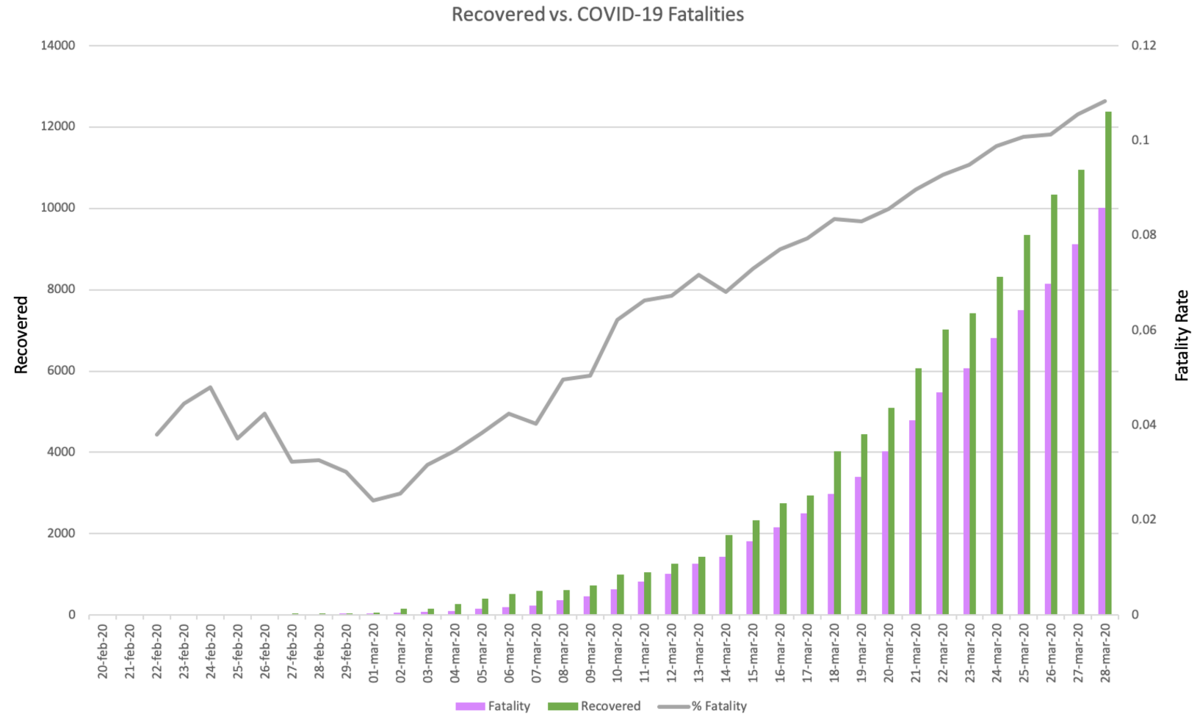
Graphical plot of COVID-19 fatalities and recovered in Italy.

Additionally, in that case, it is important to observe that the curve of fatalities and recoveries (as depicted in Figure 7) were misaligned temporally with the curve of new cases, since fatalities occur on average after 8 days, while recoveries are an additional 14 days, and new cases are observed after 8 days of COVID-19 incubation (on average). This explains why the peak in the number of new cases had been reached, while the peak on daily fatalities was approaching, and we expected it at the end of March. The same is true for the peak of recoveries that we expected to appear in 2 more weeks (middle or end of April).

## Discussion

This study was conducted in the early days of the pandemic in Italy to promptly define a model able to predict the curve of total positive cases in Italy and the Lombardy Region.

The model predicted the real data published daily by the Department of the Italian Civil Protection, estimating in a precise manner and several months in advance the plateau for both the logistic curves for the Lombardy Region and Italy, and the end of this first COVID-19 pandemic wave. This suggests the possibility to generalize the model for other countries, which will follow the restrictions imposed by the Italian government, to have a clear picture on the evolution of the number of new cases and to act promptly with policies and restrictions that can maximize care and treatments offered to patients with COVID-19. Moreover, this paper shows that big data and data analytics can help medical experts and epidemiologists in promptly designing accurate models to predict the different COVID-19 evolutionary phases in other countries and regions, and for second and third possible epidemic waves.

## Abbreviations

WHO: World Health Organization
